# Polyvalent DNA Vaccines Expressing HA Antigens of H5N1 Influenza Viruses with an Optimized Leader Sequence Elicit Cross-Protective Antibody Responses

**DOI:** 10.1371/journal.pone.0028757

**Published:** 2011-12-21

**Authors:** Shixia Wang, Anthony Hackett, Na Jia, Chunhua Zhang, Lu Zhang, Chris Parker, An Zhou, Jun Li, Wu-Chun Cao, Zuhu Huang, Yan Li, Shan Lu

**Affiliations:** 1 Jiangsu Province Key Laboratory in Infectious Diseases, The First Affiliated Hospital of Nanjing Medical University, Nanjing, China; 2 Department of Infectious Diseases, The First Affiliated Hospital of Nanjing Medical University, Nanjing, China; 3 China-US Vaccine Research Center, The First Affiliated Hospital of Nanjing Medical University, Nanjing, China; 4 Beijing Institute of Microbiology and Epidemiology, and State Key Laboratory of Pathogen and Biosecurity, Beijing, China; 5 National Microbiology Laboratory, Public Health Agency of Canada, Winnipeg, Manitoba, Canada; 6 Department of Medicine, University of Massachusetts Medical School, Worcester, Massachusetts, United States of America; University of Georgia, United States of America

## Abstract

Highly pathogenic avian influenza A (HPAI) H5N1 viruses are circulating among poultry populations in parts of Asia, Africa, and the Middle East, and have caused human infections with a high mortality rate. H5 subtype hemagglutinin (HA) has evolved into phylogenetically distinct clades and subclades based on viruses isolated from various avian species. Since 1997, humans have been infected by HPAI H5N1 viruses from several clades. It is, therefore, important to develop strategies to produce protective antibody responses against H5N1 viruses from multiple clades or antigenic groups. In the current study, we optimized the signal peptide design of DNA vaccines expressing HA antigens from H5N1 viruses. Cross reactivity analysis using sera from immunized rabbits showed that antibody responses elicited by a polyvalent formulation, including HA antigens from different clades, was able to elicit broad protective antibody responses against multiple key representative H5N1 viruses across different clades. Data presented in this report support the development of a polyvalent DNA vaccine strategy against the threat of a potential H5N1 influenza pandemic.

## Introduction

The continuous spread of highly pathogenic avian influenza Type A (HPAI) H5N1 viruses in avian species across multiple continents and frequent reports of human H5N1 infection in China and Southeast Asia highlight the threat of a potential flu pandemic in the human population. At the same time, H5N1 viruses have grown into genetically and antigentically diversified viruses. Based on phylogenetic analysis of hemagglutinin (HA) protein gene sequences, at least 10 clades of H5N1 viruses (clades 0–9) have been identified [1], [2], [3], [4], [5]. Recent studies have further assigned these viruses into four major antigenic groups (A–D) [3]. HPAI H5N1 viruses from more than one clade have caused human infection since 1997.

A key component in the global strategy to prepare for and control any pending influenza pandemic is the development of an effective vaccine. Several versions of inactivated as well as live attenuated H5N1 vaccines have been tested in humans and showed an overall good safety and immunogenicity profile mainly by using a clade 1 H5N1 virus (A/Vietnam/1203/04) as the vaccine strain per recommendations by the World Health Organization (WHO) [6], [7], [8]. Given that the majority of the world's human population is naïve to H5N1 influenza, two immunizations are needed to achieve desired levels of protective immune responses against H5N1 in contrast to the annual seasonal flu vaccine which requires only one immunization, presumably due to the priming effects by either exposure to circulating H1, H3 or Type B influenza viruses in humans or history of prior seasonal flu vaccination. The likely requirement of two immunizations in conjunction with the genetic complexity of H5N1 viruses, as evidenced by their separation into multiple subgroups, makes it difficult to prepare for the timely production of a sufficient number of doses of H5N1 vaccines in the event of an H5N1 pandemic; therefore, supplemental strategies are needed. As shown by our previously published report [9] and confirmed by other recent studies [10], a DNA prime-inactivated vaccine boost is highly effective in eliciting higher protective immune responses than using either DNA or inactivated flu vaccine alone. Therefore, it may be possible to use DNA vaccines as the first dose of immunization that can be given either long before the pandemic (pre-pandemic vaccination) or shortly after the outbreak, to reduce the burden on the production of inactivated vaccines at the time of the outbreak. Furthermore, DNA vaccines can be stockpiled for a long period of time, which makes this method even more attractive.

One key issue that needs to be analyzed for the above strategy is the cross reactivity between DNA vaccines expressing H5 HA antigens from different clades. It is critical to first optimize the immunogenicity of H5 HA DNA vaccines and then to test how much cross protection can be achieved with optimized H5 HA DNA vaccines. In the current report, we constructed DNA vaccines to express wild type HA antigens without mutations at the HA1 and HA2 cleavage site from four key H5N1 strains that have caused major human infection: HK/156/97 (clade 0), VN/1203/04 (clade 1), Ind/5/05 (clade 2.1), and Anhui/1/05 (clade 2.3). Rabbit sera immunized with these HA antigens were examined for their protective antibody responses against either homologous or heterologous H5N1 viruses. Our results demonstrated an imperfect cross-reactivity profile for the protective antibody responses among these four viruses. A polyvalent formulation including three different H5 HA DNA vaccines was able to produce broad protective antibody responses with high titers against these key H5N1 isolates. Information learned from this study should facilitate the selection of candidate H5N1 vaccines to form polyvalent H5N1 DNA vaccines as part of the global strategy to prevent and control a potential avian flu pandemic.

## Results

### Designs of DNA vaccines expressing different forms of H5 HA antigens

**Figure 1:**
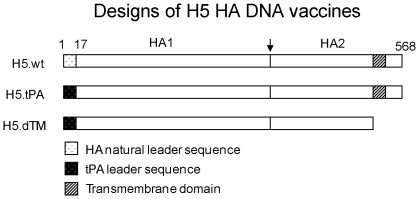
Schematic diagram of influenza A H5 HA gene inserts used in codon optimized DNA vaccines (A/HongKong/156/97 and A/VietNam/1203/2004), including the full length HA antigens with natural leader sequence (H5.wt) or a human tissue plasminogen activator (tPA) leader substituting the natural HA leader sequence (H5.tPA) and the transmembrane/cytoplasmic region truncated HA antigens (H5.dTM) with tPA leader sequence. The cleavage site between HA1 and HA2 subunits are marked. The numbers above the HA inserts denote the relevant amino acid positions in natural HA proteins.

One of the key findings from our previous study was that HA antigens from H1 and H3 serotypes had different structure preferences in order to elicit optimal protective antibodies [11]. Two of the HA antigen designs used in that study were also included in the current study to identify the optimal design for the H5 serotype HA antigens: one used the wild type HA antigen insert (H5.wt), which has the exact same amino acid sequences found in the natural viral isolate, and the other used a truncated HA antigen insert (H5.dTM), which removed the transmembrane (TM) and intracellular segments of the HA2 domain (Fig. 1). In addition, a third HA antigen insert was created (H5.tPA), in which a human tissue plasminogen activator (tPA) sequence replaced the original wild type leader sequence from the HA antigen (Fig. 1). The third HA antigen design was adopted because the H5.dTM design also used a tPA leader sequence and the H5.tPA insert served as a control for the H5.dTM insert to understand the role of the tPA leader when it is incorporated as the only change in the design from the original wild type HA antigen insert.

In order to maximize the immunogenicity of HA DNA vaccines as shown in previous studies [9], [11], HA genes used in the current study were also codon optimized and chemically synthesized. In addition, the HA gene sequences used in the current study express intact HA amino acid sequences at the cleavage site between HA1 and HA2 (PQREXRRKKR↓G) of HA proteins in highly pathogenic H5N1 viruses [12], [13], [14]. This cleavage was shown to be important for the pathogenesis of H5N1 viruses [12], [15]. For inactivated H5 serotype flu vaccines, these residues were removed to improve the safety profile of such vaccines for both manufacturing and mass immunization purposes [7] [16], [17], [18].

### Expression and immunogenicity of different forms of DNA vaccines coding for the HA antigen of a 1997 H5N1 influenza Hong Kong isolate

**Figure 2:**
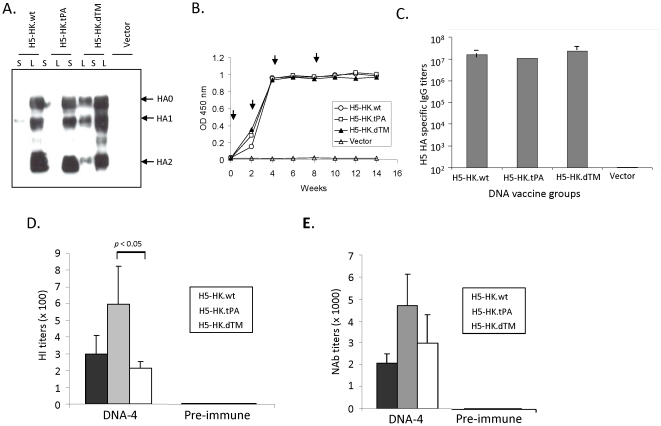
Antigen expression and immunogenicity of H5-HK HA DNA vaccines. A. Western blot analysis of the expression, secretion, and susceptibility to cleavage expressed by different H5-HK (A/HongKong/156/97) HA DNA vaccine constructs in transiently transfected 293T cell supernatant (S) and cell lysate (L). B. Temporal serum anti-HA IgG responses in rabbits measured by ELISA at 1∶5000 serum dilution against H5-HK-dTM as coating antigen. The arrows indicate the time of gene gun-mediated DNA immunizations. Each curve represents the OD value of each group of 3 rabbits receiving H5-HK.wt, H5-HK.tPA, H5-HK.dTM DNA vaccine or empty DNA vector as indicated. C. End titration titers of serum anti-H5-HK HA IgG responses at two weeks after the 4^th^ DNA immunization from the same rabbits shown in panel B. D. The hemagglutination inhibition (HI) antibody responses in NZW rabbit sera immunized with different designs of H5-HK HA DNA vaccines collected at 2 weeks after the 4^th^ DNA immunization (DNA-4) or relevant pre-bleed sera as indicated. The HI antibody titers are shown as the geometric means for each group (3 rabbits per group) with standard deviations against H5N1 A/HongKong/483/97 virus. E. Neutralizing antibody (NAb) responses in NZW rabbit sera immunized with different designs of H5-HK HA DNA vaccines collected at 2 weeks after the 4^th^ DNA immunization (DNA-4) or relevant pre-bleed sera as indicated. NAb titers against H5N1 A/HongKong/483/97 virus infection to MDCK cells are shown as the geometric means from each group (3 rabbits per group) with standard deviation. The statistical differences between each testing groups are determined and P values less than 0.05 or 0.01 are indicated.

The first set of H5 HA DNA vaccines was produced by cloning codon optimized HA genes based on the amino acid sequences of HA antigen from an H5N1 influenza isolate A/HongKong/156/97, which was responsible for the first outbreak of H5N1 avian influenza in humans in 1997, into a DNA vaccine vector [19]. The expression of HA antigens from three different H5-HK DNA vaccines was examined in transiently transfected 293T cells (Fig. 2A). Regardless of whether the natural HA leader or tPA leader was used, HA proteins expressed from two full length H5-HK DNA vaccine constructs (HA-HK.wt and HA-HK.tPA) were detected in cell lysate but not in the supernatant, suggesting they are mainly cell-associated. In contrast, truncation of the C-terminal segment, including the removal of the TM domain in the H5-HK.dTM DNA vaccine construct, was able to significantly increase the secretion of HA protein (detected in supernatant) (Fig. 2A). H5-HK HA proteins expressed by all of three HA DNA vaccine designs were able to be cleaved into HA1 and HA2 subunits (Fig. 2A).

New Zealand White (NZW) rabbits were used in the current study to produce large quantities of sera for both binding antibody and functional antibody analyses. Animals were immunized with one of the three H5-HK HA DNA vaccines (individually) via gene gun. Positive antibody responses were elicited in immunized rabbit sera against the HK HA antigen and levels of such responses increased with repeated immunizations while the negative control rabbit group that received empty DNA vector did not have HA-specific antibody responses (Fig. 2B). As measured by both temporal and peak-level antibody responses, there was no difference in the ability of the three forms of H5-HK HA DNA vaccines to elicit H5 HA-specific antibody responses (Fig. 2B and 2C).

However, functional antibody analyses with the rabbit anti-HA immune sera showed a very different picture when these sera were further analyzed by either the hemagglutination inhibition (HI) or microneutralization (MN) assays. All three forms of H5-HK HA DNA vaccines induced protective antibody responses against the autologous wild type virus A/HongKong/483/97, but levels of protective antibodies were different among sera induced by different designs of H5-HK HA DNA vaccines. The DNA vaccine with the full length HA insert under the tPA leader sequence (H5-HK.tPA) elicited consistently higher HI and MN antibody titers when compared to the other two forms of HK-HA inserts, the full length HA with a natural leader sequence (H5-HK.wt), and the transmembrane region (TM) truncated HA (H5-HK.dTM). The difference was statistically significant (p<0.05) between H5-HK.tPA and H5-HK.dTM sera based on the HI assay (Fig. 2D) and between H5-HK.tPA and H5.HK.wt sera based on the MN assay (Fig. 2E).

### Expression and immunogenicity of different forms of DNA vaccines coding for the HA antigen of an H5N1 isolate A/VietNam/1203/04

**Figure 3:**
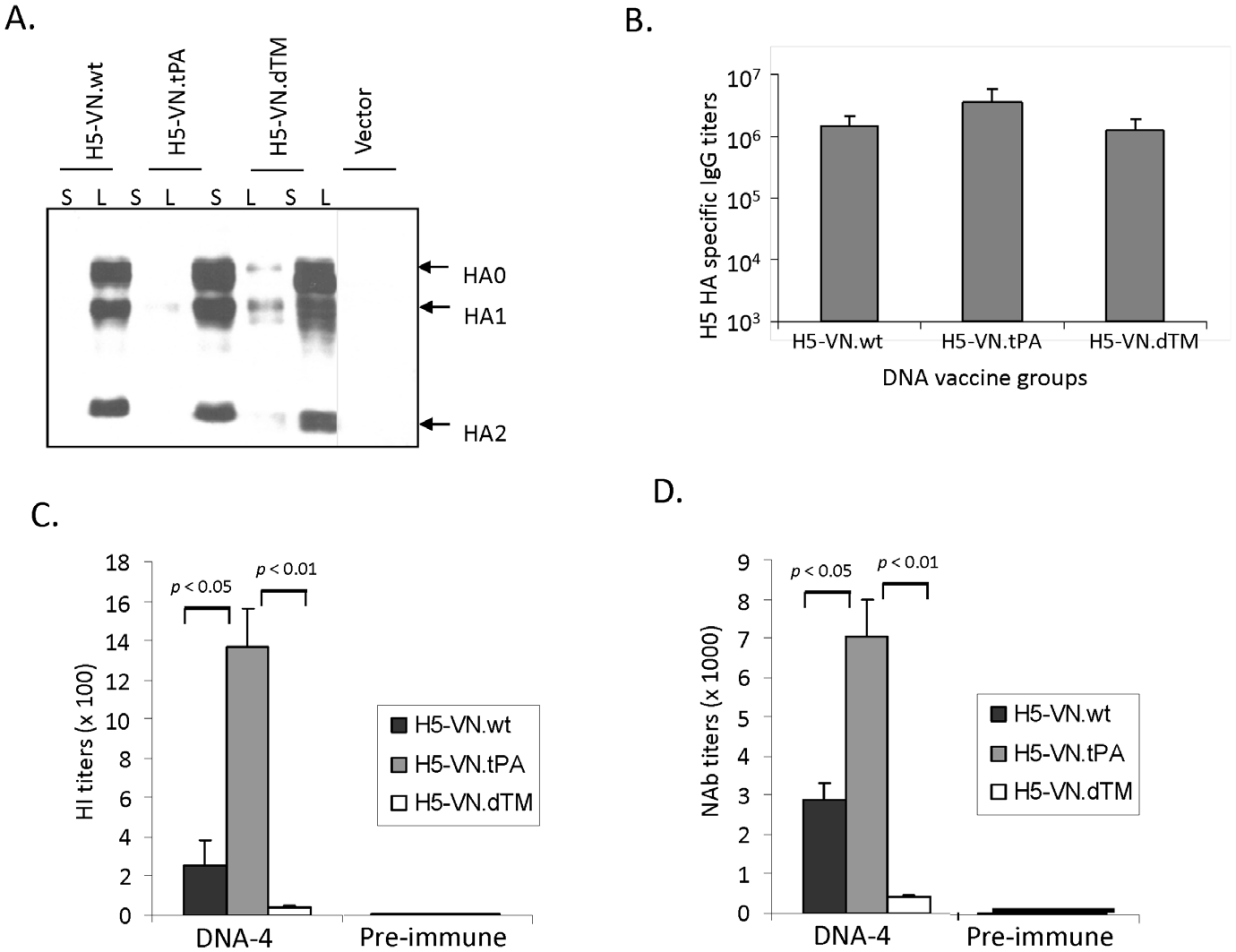
Antigen expression and immunogenicity of H5-VN HA DNA vaccines. A. Western blot analysis of the expression, secretion, and susceptibility to cleavage expressed by different H5-VN (A/VietNam/1203/2004) HA DNA vaccine constructs in transiently transfected 293T cell supernatant (S) and cell lysate (L). B. End titration titers of serum anti-H5-VN HA IgG responses at two weeks after the 4^th^ DNA immunization from rabbits immunized with differently designed H5-VN DNA vaccines against H5-VN.dTM as coating. C. The hemagglutination inhibition (HI) antibody responses in NZW rabbit sera immunized with different designs of H5-VN HA DNA vaccines collected at 2 weeks after the 4^th^ DNA immunization (DNA-4) or relevant pre-bleed sera as indicated. The HI antibody titers are shown as the geometric means for each group (3 rabbits per group) with standard deviations against H5N1 A/VietNam/1203/04 virus. D. Neutralizing antibody (NAb) responses in NZW rabbit sera immunized with different designs of H5-VN HA DNA vaccines collected at 2 weeks after the 4^th^ DNA immunization (DNA-4) or relevant pre-bleed sera as indicated. NAb titers against H5N1 A/VietNam/1203/04 virus infection to MDCK cells are shown as the geometric means from each group (3 rabbits per group) with standard deviation. The statistical differences between each testing groups are determined and P values less than 0.05 or 0.01 are indicated.

In order to rule out that the above finding was not only unique to this H5N1 virus isolate from Hong Kong in 1997, similar designs of HA inserts were produced by using a codon optimized HA DNA gene from the H5N1 strain A/VietNam/1203/04, a well-studied representative isolate for the H5N1 viruses [20], [21]. The pattern of H5-VN HA expression was similar to that of H5-HK HA antigens. Only cell-associated HA antigens were detected with H5-VN.wt and H5-VN.tPA constructs in contrast to that identified with the H5-VN.dTM, which had HA antigen expression in both cell lysate and supernatant fractions (Fig. 3A). Rabbits were immunized with the electroporation method as previously reported [22]. Similar to H5-HK DNA plasmids, binding antibody responses, as measured by ELISA, showed similar levels among sera elicited by the three H5-VN DNA vaccines with different HA gene insert designs (Fig. 3B). However, functional antibodies, as measured by HI and MN antibody analyses, revealed again that the H5-VN.tPA design induced the highest levels of functional antibody responses (Fig. 3C and 3D). In the case of functional antibody responses against the wild type virus A/VietNam/1203/04, the differences between H5-VN.tPA and the other two forms were statistically significant by both HI and MN assays (p<0.05 or p<0.01).

**Figure 4:**
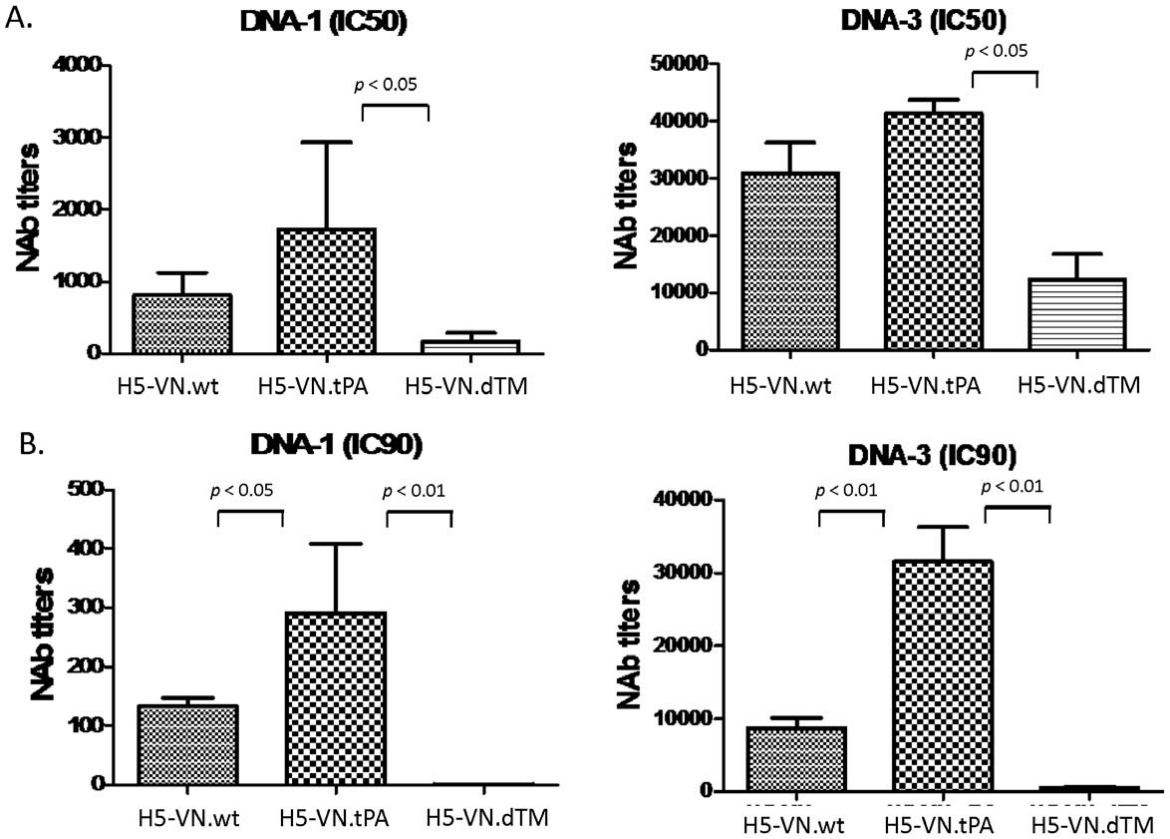
Neutralizing antibody responses detected by the pseudotyped virus system expressing the H5 VN-HA antigen. Early time point sera, at 2 weeks after either one (DNA-1) or three (DNA-3) DNA immunizations, were collected from the same groups of rabbits included in Fig. 3. A. Neutralizing antibody titers measured at 50% inhibition (IC50) of virus infection to target cells. B. Neutralizing antibody titers measured at 90% inhibition (IC90) of virus infection to target cells. Data shown are the geometric mean titers of each group with standard deviations. The statistical differences between each testing groups are determined and P values less than 0.05 or 0.01 are indicated.

In order to ensure that the difference in protective antibody responses between sera elicited by H5-VN.wt and H5-VN.tPA was not the result of repeated immunizations, sera collected after one or three immunizations were also measured (Fig. 4). Pseudotyped viruses expressing VN HA antigen were used to measure the neutralizing antibody activities in rabbit sera with less immunizations. The strength of protective antibodies was measured at two levels: inhibition concentrations that can block either 50% (IC50) or 90% (IC90) of virus infection to target cells. Both measurements showed that H5-VN.tPA-elicited rabbit sera had significantly higher titers of neutralizing antibody activities than the wild type H5 HA design, especially when using the more stringent IC90 as a cut-off (p<0.05 or p<0.01) (Fig. 4).

### Sensitivity to deglycosylation treatment for HA antigens expressed by different forms of H5-VN HA DNA vaccines

Additional studies were conducted to ask if glycosylation of HA has been affected with the use of a different leader sequence which may influence the immune responses. Our previous study with a hepatitis B surface antigen suggested that post-translational modifications including glycosylation may affect the immunogenicity of DNA vaccine delivered antigens [23]. The Asn (N)-linked glycosylation of influenza HA proteins are essential for virus infectivity and vaccine immunogenicity [24], [25]. Based on sequence analysis, there are 8 to 9 N-linked glycosylation sites (Asn-X-Ser/Thr) in H5N1 HA proteins. We next investigated whether the HA antigens expressed by different designs of H5 HA inserts in the above DNA vaccinations have similar levels of N-linked glycosylation. The HA antigens expressed in 293T cell lysate transfected with the H5-VN.wt, H5-VN.tPA or H5-VN.dTM DNA vaccines and HA antigen expressed in the 293T cell supernatant transfected with the H5-VN.dTM DNA vaccine were analyzed for their susceptibility to PNGase treatment, which can cleave any type of N-linked sugar including complex oligosaccharide structures resulting from the maturation of high mannose moieties during transport of the glycoprotein through the Golgi.

Treatment with PNGaseF allowed for the removal of N-linked glycosylations, as shown by the reduction of apparent molecular weight of HA0, HA1, and HA2 species in Western blot analysis for HA proteins expressed in cells transfected by these H5-VN HA DNA vaccines (Figure S1). The molecular weight reduction patterns for HA0, HA1, and HA2 antigens were the same between cells transfected by H5-VN.wt and H5-VN.tPA DNA vaccines, suggesting that HA antigens expressed by these two HA DNA vaccines were similarly glycosylated. A smaller molecular weight HA2 antigen was observed in cells transfected with the H5-VN.dTM DNA vaccine, presumably due to the truncated size of HA2 domain in this particular HA insert design; however, PNGaseF treatment also led to a proportional reduction of molecular weight for truncated HA2 protein in both supernatant and cell lysate preparations. The only unique finding is that the cell-associated HA antigens in H5-VN.dTM transfected cells showed a high level of heterogeneity and a small portion of the HA1 proteins was not fully deglycosylated by PNGaseF treatment, reflecting the continued presence of different forms of glycosylated HA proteins in H5-VN.dTM transfected cells. Otherwise, the overall glycosylation pattern, as probed by deglycosylation treatment, was very similar among HA antigens produced by three different types of H5 HA DNA vaccines.

### Cross-protective antibody responses induced by individual DNA vaccines expressing HA antigens from key H5N1 viral isolates

Based on the above results, additional HA DNA vaccines with the HA.tPA insert design were produced by using codon optimized HA genes that encode the HA proteins from H5N1 viral strains A/Anhui/1/2005 and A/Indonesia/5/2005, both have caused human infection in recent years [26], [27]. Given the circumstance that H5N1 influenza antigen drifts have occurred since the first human outbreak in Hong Kong in 1997, it would be important to determine if H5 HA vaccines developed based on H5N1 viruses isolated at different epidemic time points can induce cross antibody responses against other H5N1 viruses.

One set of experiments was conducted to understand the cross protection between paired H5N1 HA antigens. Rabbits were immunized with individual H5-HK.tPA, H5-VN.tPA, and H5-AH.tPA DNA vaccines and rabbit immune sera were examined for HA antigen-specific antibody responses. H5 HA-specific antibody responses against H5 HA antigens from different viruses were analyzed by ELISA and the potential cross-protective antibody responses against different H5N1 viruses were evaluated by HI and MN assays.

**Figure 5:**
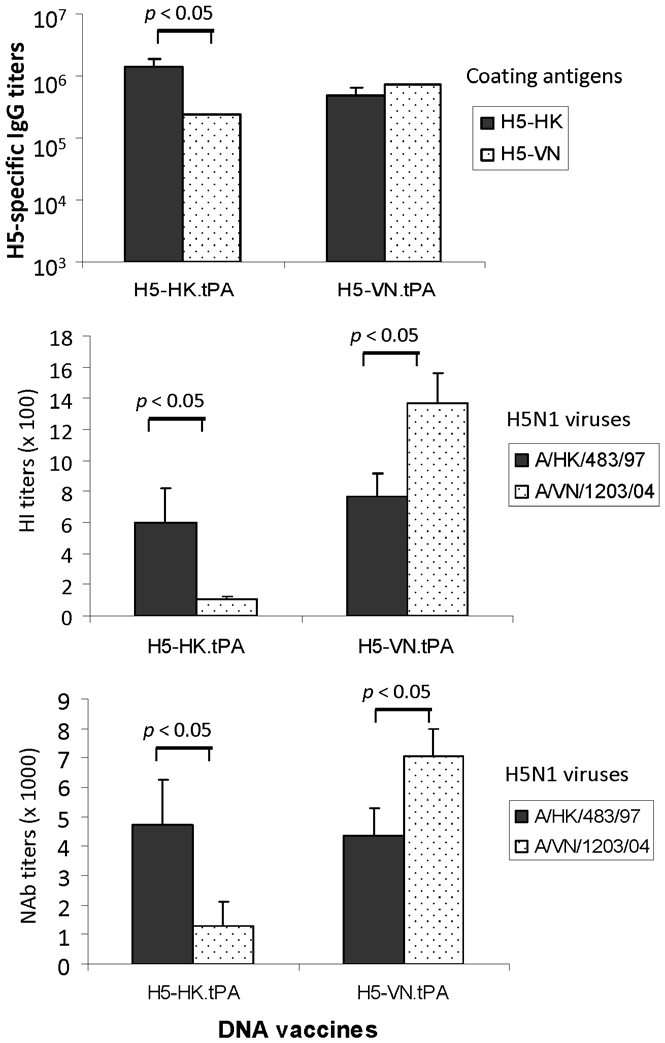
Levels and specificity of anti-HA IgG (upper panel), hemagglutination inhibition (HI, middle panel), and neutralizing (NAb, lower panel) antibody responses elicited by H5-HK and H5-VN HA DNA vaccines in rabbits immunized with H5-HK-tPA and H5-VN-tPA (3 rabbits/group), respectively, at two weeks after the 4^th^ DNA immunization. Peak serum level anti-H5 HA IgG responses as measured by ELISA against H5-HK-dTM or H5-VN-dTM antigens. HI antibody titers and NAb were detected against H5N1 A/HongKong/483/97 or A/VietNam/1203/04 virus, respectively. Neutralizations against wild type H5N1 viruses were performed in MDCK cells.

H5-HK (A/HK/156/97), a clade 0 H5N1 isolate, and H5-VN (A/VN/1203/04), a clade 1 H5N1 isolate, represent H5N1 viruses isolated from the first human outbreak in Hong Kong in 1997 and a subsequent outbreak in Vietnam in 2004, respectively. Results shown in Fig. 5 indicate that H5-HK.tPA DNA vaccine-immunized rabbit sera showed high antibody responses recognizing both the autologous H5-HK HA antigen and the heterologous H5-VN HA antigen. However, the HA-specific IgG titers against the autologous H5-HK antigen were higher than those observed against the heterologous H5-VN antigen (p<0.05). Conversely, the H5-VN.tPA DNA vaccine elicited high level HA-specific IgG responses against autologous H5-VN and heterologous H5-HK HA antigens although the overall titers against its autologous H5-VN HA antigen may be higher (not statistically significant).

Protective HI and MN antibody responses induced by H5-HK.tPA and H5-VN.tPA DNA vaccines were further compared against either A/HK/483/97 or A/VN/1203/04 wild type viruses (Fig. 5). HI and MN titers were present in both HK.tPA and H5-VN.tPA DNA vaccine-immunized rabbit sera against both viruses, but such protective antibody responses showed a degree of strain specificity. Statistically significant higher HI and MN titers were observed in H5-HK.tPA DNA vaccine-immunized rabbit sera against the autologous virus, A/HK/483/97, when compared to responses observed against the heterologous virus, A/VN/1203/04 (p<0.05). Similarly, the H5-VN.tPA DNA vaccine induced much higher HI and MN responses against its autologous virus, A/VN/1203/04, compared to the heterologous virus, A/HK/483/97 (p<0.05).

**Figure 6:**
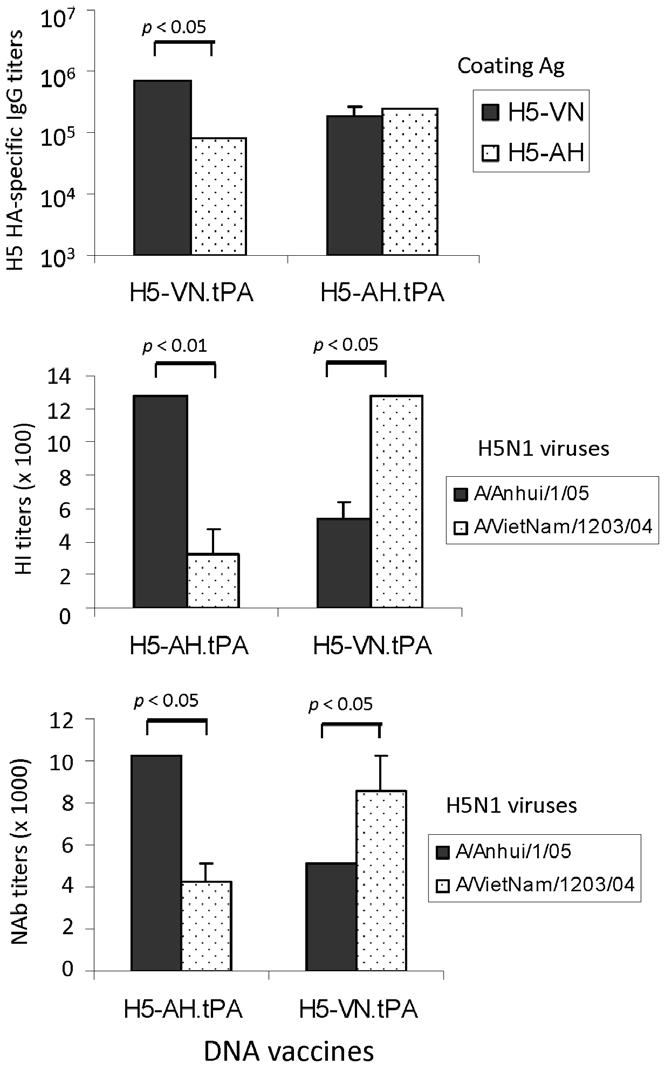
Levels and specificity of anti-HA IgG (upper panel), HI (middle panel), and NAb (lower panel) antibody responses elicited by H5-VN (A/VietNam/1203/2004) and H5-AH (A/Anhui/1/2005) HA DNA vaccines in rabbits immunized with H5-VN-tPA (3 rabbits) and H5-AH-tPA (4 rabbits), respectively, at two weeks after the 4^th^ DNA immunization. Peak serum level anti-H5 HA IgG responses, as measured by ELISA against the H5-VN-dTM or H5-AH-dTM antigens. HI antibody and NAb titers were detected against H5N1 A/VietNam/1203/04 or A/Anhui/1/2005 virus, respectively. Neutralizations against wild type H5N1 viruses were performed in MDCK cells. Data shown are the geometric mean IgG, HI, or NAb titers of each group with standard deviations. The statistical differences between each testing groups are determined and P values less than 0.05 or 0.01 are indicated.

Similar analysis was conducted with rabbit immune sera elicited by H5-VN and H5-AH HA DNA vaccines (Fig. 6). H5-AH (A/Anhui/1/05), a clade 2.3 H5N1 isolate, represents the H5N1 virus isolated from a human outbreak in China in 2005 [26]. The cross reactivity between H5-VN and H5-AH immune sera and viruses was very similar to that observed above between H5-HK and H5-VN immune sera and viruses. For binding antibody responses, H5-VN rabbit immune sera had significantly higher recognition to its autologous H5-VN HA antigen than the heterologous H5-AH HA antigen (p<0.05). H5-AH rabbit immune sera elicited higher antibody responses recognizing the autologous H5-AH HA antigen than the heterologous H5-VN HA antigen (not statistically significant) (Fig. 6). For functional antibodies, both HI and MN analyses showed preference for H5-VN and H5-AH rabbit immune sera against their respective autologous wild type viruses, A/VN/1203/04 and A/Anhui/1/05 (p<0.05 or p<0.01) (Fig. 6).

### Cross-protection by a polyvalent DNA vaccine formulation expressing HA antigens from three representative H5N1 viral isolates

The above results indicate that while antibody responses against one H5N1 HA antigen or virus may well cross-react with another H5N1 HA antigen or virus, the levels of antibody responses against the autologous antigen or virus were always higher. Given the uncertainty regarding which H5N1 virus may ultimately cause a pandemic H5N1 outbreak, it is important to develop a vaccine strategy that can maximize the protection efficacy against a wide spectrum of H5N1 viruses from different clades (or subtypes). It is possible that a consensus HA antigen, or a structurally-optimized HA antigen design, can cover different H5N1 viruses at various levels of protective efficacy but there is no actual data showing that such HA antigen can achieve the maximum protective antibody responses against several H5N1 viral isolates from different clades.

One alternative approach is to produce a polyvalent HA formulation by including multiple H5N1 HA DNA vaccines in one injection to produce an immune sera that can induce the highest antibody response against a wide range of H5N1 viral isolates. For this purpose, we have tested one such polyvalent HA DNA vaccine formulation by injecting rabbits with three H5 HA DNA vaccines: H5-VN.tPA, H5-AH.tPA, and H5-IN.tPA DNA vaccines. A H5-IN.tPA DNA vaccine was produced expressing the HA antigen from another key H5N1 viral isolate, A/Indonesia/5/2005, a clade 2.1 H5N1 isolate. Rabbits in other groups received only one H5 HA DNA vaccine (monovalent). Functional antibody analysis was conducted against pseudotyped viruses expressing different individual H5 HA and N1 antigens.

**Figure 7:**
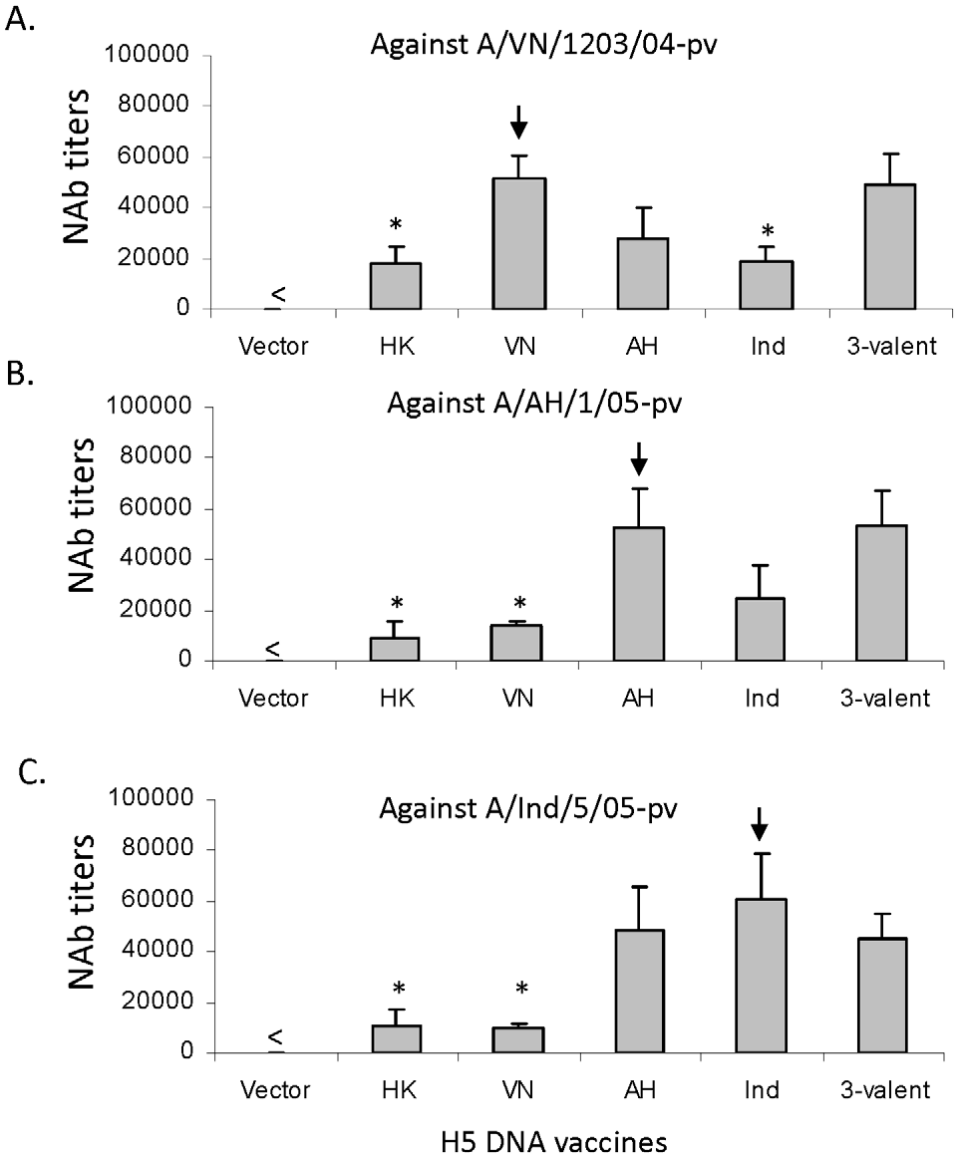
Levels and specificity of NAb responses against H5 pseudotyped viruses expressing full length H5 HA from A/VietNam/1203/2004 (A/VN/1203/04-pv, Panel A), A/Anhui/1/2005 (A/AH/1/05-pv, Panel B) or A/Indonesia/5/2005 (A/Ind/5/05-pv, Panel C), by different H5-HA DNA vaccines or empty DNA vector in rabbits. The vaccine group “HK”, “VN”, “AH” or “Ind” represent monovalent H5 DNA vaccines HK.tPA, VN.tPA, AH.tPA, or Ind.tPA, respectively. The “3-valent” group consists of VN-HA.tPA AH-HA.tPA and Ind-HA.tPA H5 DNA vaccines. There were 3 rabbits/group in Vector, HK, VN, AH, and Ind groups, and 4 rabbits/group in 3-valent groups. Rabbit sera tested by the pseudotyped NAb assays were collected at 2 weeks after the 4^th^ DNA immunization. “<” denotes below detection level. The arrow denotes neutralization against the autologous H5 pseudotyped virus. The statistical differences between the testing group and the autologous neutralization group or 3-valent group are indicated by “*” when the p value was less than 0.05.

The pseudotyped virus system, developed in recent years and widely used in leading influenza studies, provides high sensitivity in detecting functional antibody responses against influenza HA antigens, and at the same time, eliminates the influence of other influenza viral gene products since a common viral backbone is used for different HA pseudotyped viruses [28], [29]. It is an ideal system as a high-throughput assay for multiple serum samples against a wide range of viruses. As shown in Fig. 6, rabbit sera elicited by either the 3-valent HA DNA vaccine formulation (H5-VN+H5-AH+H5-IN) or the monovalent H5 HA DNA vaccines (HK, VN, AH, and IN) were tested for their antibody responses against three pseudotyped viruses expressing H5-VN, H5-AH, or H5-IN HA antigens. The matched monovalent rabbit sera consistently showed the highest functional antibody responses against autologous pseudotyped viruses (Fig. 7A–7C). However, the 3-valent serum was the only one that showed high level antibody responses against all three pseudotyped viruses while one non-matched monovalent rabbit serum could neutralize one or two viruses but not all three, further confirming the hypothesis that a polyvalent HA formulation is capable of protect against multiple H5N1 viruses.

## Discussion

According to phylogenetic analysis, H5N1 viruses can be divided into 10 clades (0–9). Since 1997, humans have mainly been infected by H5N1 viruses from clades 0, 1, and 2, although there have also been reports of infection by clade 7 virus [5]. Clade 2 is the most complicated in its genetic evolution and has been further divided into five subclades (2.1 to 2.5). In the current study, we have selected HA antigens from one clade 0 virus (A/HongKong/156/97), one clade 1 virus (A/VietNam/1203/2004), two clade 2 viruses (A/Indonesia/5/2005, clade 2.1; and A/Anhui/1/2005, clade 2.3) [3].

While progress has been made in reducing the number of required immunizations during vaccination with inactivated H5N1 vaccines by incorporating various adjuvants into the vaccine formulations, a major next-step for H5N1 vaccine research is to determine to what degree the immunity elicited by one H5 avian influenza vaccine (currently, many candidate H5N1 vaccines were developed based on a clade 1 virus (A/VietNam/1203/2004)) can cross-protect against H5N1 viruses from other clades. Unlike human Type A influenza viruses (H1 or H3 serotypes), any potential pandemic caused by an H5N1 virus will be of avian origin and, in theory, any of the current known H5N1 avian viruses may jump to the human population leading to the next pandemic. Therefore, a systemic examination on the cross-protection among HA antigens from different clades is needed for strategic planning to determine whether more than one H5N1 vaccine is needed based on the analysis of protection profiles, and if so, what particular viral strains should be selected to provide the maximum breadth of protection.

DNA vaccination is an attractive strategy to provide relatively quick and straightforward production of vaccines against an influenza pandemic when the demand for such vaccines suddenly increases. However, a key issue surrounding the use of DNA vaccines is their low immunogenicity in humans. In recent years, the success of the prime-boost strategy has greatly enhanced the utility of DNA vaccination for future human applications [10], [30]. At the same time, optimization of the design of antigen inserts based on the uniqueness of each antigen (a process of “antigen engineering”) [31], [32] can also play a key role.

Results included in the current report indicated that the tPA leader sequence and the C-terminal transmembrane domain/cytoplasmic region of H5 HA both contribute to better functional antibody responses in H5.tPA DNA vaccines when compared to H5.wt and H5.dTM DNA vaccines. The above findings were different from our previous results on protective antibody responses induced by differently designed flu H1 and H3 HA DNA vaccines [11]. In this previous study, only the full length H1.wt but not transmembrane truncated H1.dTM induced high level HI and MN responses against H1 virus while both the full length H3.wt and truncated H3.dTM induced similar HI and MN responses against H3 virus [11]. These results provide a strong indication that the HA antigens from different influenza A subtypes (H1, H3 and H5) may have different preferences for antigen structure designs in order to generate optimal protective antibody responses.

Studies were conducted in this report to identify the mechanism responsible for better protective antibodies in rabbit immune sera elicited by the H5.tPA HA insert design but the exact mechanism is currently unclear. First, we tested whether a higher level of HA antigen expression was produced with the tPA-leader design. As shown in Fig. 2A and 3A, antigen expression levels between WT-leader design and tPA-leader design were similar, thus excluding this possibility. Next, we asked whether there is increased secretion of the HA antigen due to the use of the tPA leader. However, as shown in Fig. 2A and 3A, there is no major detectable level of secreted HA antigens in supernatant for either the WT-design or tPA-design. Furthermore, the dTM design did have a higher level of secretion due to the deletion of transmembrane and intracellular portion of HA protein but did not elicit better protective antibody responses. Finally, a study was conducted to examine the possible role of post-translational processing, such as a change in glycosylation, of HA the antigen, which may affect the antigen processing pathway, as we previously reported with a hepatitis B surface antigen DNA vaccine [33], [34], [35]. However, Figure S1 showed that there is no major difference between WT-design and tPA-design after de-glycosylation treatment.

Therefore, it is very likely that HA antigen expressed with the tPA leader may be more effective in eliciting conformational antibodies. This hypothesis is supported by two pieces of evidence. First, there was no difference in the levels of binding antibodies as measured by ELISA, which indicated that there was no difference in the general immunogenicity between WT and tPA leader designs; only a difference in the functional antibody was observed. Second, the HA.dTM DNA vaccine design also used the tPA leader but did not have better functional antibodies, proving that the proper folding of the HA antigen in the presence of a tPA leader is important and is dependent on the presence of an intact HA2 domain. It is possible that such an HA antigen conformation is part of a trimer structure of HA since the HA2 domain is involved in the formation of HA trimers. Only antibodies against the trimer structure are more functionally relevant to block the trimer form of HA spikes on viral particles.

By using the optimal H5.tPA HA insert design, studies in this report further demonstrated that there are good levels of cross protection by one H5 HA DNA vaccine against multiple H5N1 viruses from different clades. It is well documented that cross protection among H5N1 viruses can be detected [33], [34], [35]. Clade 1 H5N1 vaccines (VN) cross protect against both clade 1 and clade 2 (Indonesia) viruses in ferrets with the use of a strong adjuvant [36]. Using live attenuated cold adapted (ca) viruses expressing HA and NA from 1997, cross protection was observed against the late H5 virus from 1997 to 2005 [37] and ferrets [37] and ferrets [38] and mice [39], [40] and mice [39], [40].

However, as shown in the current study, not all H5 HA vaccines can elicit the same levels of cross protective antibodies, and more significantly, maximum levels of protective antibodies were usually detected against the autologous viral isolates. Given the knowledge that protective anti-flu antibody responses in humans are much lower than in experimental animals, cross protection may not be very high in humans with one randomly selected H5 HA vaccine. In the current set of studies, it was encouraging to observe that the polyvalent H5 HA DNA vaccine was able to elicit high level protective antibody responses against multiple key H5N1 viruses. Such a polyvalent flu DNA vaccine can be used for stockpiling against a potential H5N1 pandemic even before information is available on which viral isolates may cause a human outbreak. At the same time, there are alternative approaches including the use of consensus HA antigen designs to achieve a broad coverage of various viral strains (personal communication with David Weiner). It will be interesting to compare the relative efficacy between polyvalent and consensus HA DNA vaccines in their abilities to elicit protective antibody responses. One unique advantage of the polyvalent formulation is its flexibility; one alternative HA antigen can replace or be added to the earlier polyvalent formulation in the event that a new strain of virus becomes a threat while it will be necessary to re-design the whole consensus HA insert in order to allow for broader coverage.

While HI titers against heterologous virus (cross-clade) were significantly reduced compared to HI titers against homologous virus in the current study, the heterologous virus titers were generally above 1∶100, and in humans, a HI titer of 1∶40 has been associated with protection [41], and so all of the constructs might be protective after multiple immunizations. At the same time, the titers observed in mice may not be the same as in humans. While HI titers have been associated with protection, H5 HA DNA vaccines described in the current report were not tested for protection against challenge and so there is the possibility that only some of these constructs may not protect against clinical disease or lethal infection.

No matter the design of DNA vaccines that may be used, recent studies have indicated that DNA priming immunization is effective as part of the prime –boost strategy for flu vaccine applications. In addition to DNA prime-inactivated flu vaccine boost [9], [10], a study published in 2011 further demonstrated that DNA prime-live attenuated flu vaccine boost was equal to or more effective than twice immunization with the live attenuated flu vaccine against the H5N1 viruses based on antibody responses and viral clearance in immunized ferrets [42]. Since live attenuated vaccines are considered the most immunogenic form of vaccines, it is impressive to observe that one time DNA prime was able to achieve the same priming effect as a live attenuated flu vaccine. In this particular study, the H5-VN.tPA DNA insert was used as part of the collaboration with the manufacturer of live attenuated H5N1 flu vaccine.

Based on the results published in the current report and other recent similar studies, H5N1 HA DNA vaccines evaluated in the current study should be included in the design of human studies to understand whether results reported here can be reproduced in humans when they are used as part of DNA prime, either individually or as part of the polyvalent HA DNA formulation. The finding from such studies will be very useful in the identification of simple yet powerful approaches to develop vaccines against major influenza pandemics.

## Materials and Methods

### Construction of codon optimized HA DNA vaccine constructs

The codon usage of HA genes from influenza A H5N1 viruses A/HongKong/156/97 (H5-HK), A/VietNam/1203/04 (H5-VN), A/Anhui/1/2005 (H5-AH) and A/Indonesia/5/2005 (H5-Ind) was analyzed with the MacVector software 7.2 against codon preference of *Homo sapiens*. The less optimal codons in HA genes were changed to the preferred codons in mammalian systems to promote higher expression of the HA proteins, as previously described [11]. These codon optimized HA genes were chemically synthesized by Geneart (Regensburg, Germany) with added restriction enzyme sites of PstI and BamHI for subcloning purpose immediately upstream of the start codon and downstream of the stop codon, respectively.

For either H5-HK or H5-VN HA DNA vaccines, three versions of codon optimized HA gene inserts were cloned into DNA vaccine vector pSW3891 [43]. For the first version, the full length H5-HK or H5-VN HA gene insert (568 aa,) with their natural HA leader sequences subcloned, individually, into the pSW3891 vector at the PstI and BamHI sites, designated as H5-HK.wt or H5-VN.wt DNA vaccine constructs. For the second version, the HA natural leader sequence (the first 15 aa at N-terminus for both H5-HK and H5-VN) was replaced by a human tissue plasminogen activator (tPA) leader sequence. The H5-HK and H5-VN HA gene inserts coding for aa 16–568 were PCR amplified from the full length codon optimized H5 HA genes using the following primers: H5-HA-opt-1 (gtcgctccgctagc GACCAGATCTGCATCGGCTAC) and H5-HA-opt-2 (agtcacggatcc TCAGATGCAGATCCGGCACTG). The individual H5-HK or H5-VN HA gene was cloned into the pSW3891 vector at the NheI and BamHI sites downstream of the tPA leader sequence and designated as H5-HK.tPA or H5-VN.tPA DNA vaccine constructs. For the third version, the HA natural leader sequence was replaced by a tPA leader sequence and the transmembrane (TM) and cytoplasmic region (37 aa at the C-terminus) of H5 and HA was deleted for both H5-HK and H5-VN. The truncated H5-HK or H5-VN HA genes were PCR amplified from the full length codon optimized H5-HK or H5-VN HA gene using primer pairs: H5-HA-opt-1 and H5-HA-opt-4 (agtcac ggatccTCACTGGTAGGTGCCCATGCTCTC), or H5-HA-opt-1 and H5-HA-opt-8 (agtcacggatccTCACTGGTAGATGCCGATGCTTTC), respectively. The truncated H5-HK or H5-VN gene insert was individually cloned into the pSW3891 vector at the NheI and BamHI sites downstream of the tPA leader sequence and designated as H5-HK.dTM or H5-VN.dTM.

For the H5-AH HA DNA vaccine, the construct with the full length HA under tPA-leader sequence was made as described above. Each individual DNA vaccine plasmid was prepared from *Escherichia coli* (HB101 strain) with a Mega purification kit (Qiagen, Valencia, CA) for both *in vitro* transfection and *in vivo* animal immunization studies.

### DNA immunization of New Zealand White (NZW) rabbits

NZW rabbits (∼2 kg body weight) were purchased from Millbrook Breeding Labs (Amherst, MA) for immunogenicity studies, and housed in the Department of Animal Medicine at the University of Massachusetts Medical School in accordance with IACUC approved protocol. The rabbits (3 rabbits/group) were immunized with a Helios gene gun (Bio-Rad) at the shaved abdominal skin as previously reported [44] with a total of 36 µg H5 HA DNA vaccine plasmid or vector control plasmid at each immunization. DNA immunizations were given at weeks 0, 2, 4, 8. Serum samples were taken prior to the first immunization and 2 weeks after each immunization for study of H5 HA-specific antibody responses.

### Ethics statement

This study was carried out in strict accordance with the recommendations in the Guide for the Care and Use of Laboratory Animals of the National Institutes of Health. The protocol was approved by the University of Massachusetts Medical School's Institutional Animal Use and Care Committee (IACUC) (Protocol: A-1674). All surgery was performed under sodium pentobarbital anaesthesia, and all efforts were made to minimize suffering.

### Western blot analysis of in vitro expressed HA antigens

Transient expression of the HA antigens from various HA DNA vaccine constructs were verified by Western blot analysis. HA DNA vaccine constructs were first transfected into the human embryonic kidney 293T cells using the calcium phosphate precipitation method. Briefly, 2×10^6^ 293T cells at 50% confluence in a 60 mm dish were transfected with 10 µg of plasmid DNA, and a total of 3 ml supernatant and 100 µl of cell lysate were harvested 72 hours later. Equal amounts of each transiently expressed HA antigen (10 ng of protein in 10 µl) were loaded for the SDS-polyacrylamide gel electrophoresis (SDS-PAGE) under denatured conditions, then transferred onto PVDF membranes (Bio-Rad, Hercules, CA). After being blocked overnight at 4°C in blocking buffer (0.2% I-block, 0.1% Tween-20 in 1× PBS), the membranes were incubated with a 1∶500 dilution of rabbit sera immunized with HA DNA vaccines for 30 min followed by washes. Then, the membranes were incubated with alkaline phosphatase-conjugated goat anti-rabbit IgG at 1∶5000 dilution for 30 min. Following washes, the signals were detected using a chemiluminescence-based Western-Light Kit (Tropix, Bedford, MA).

### Deglycosylation of H5 HA antigens

To analyze the N-linked glycosylation of H5 HA antigens expressed by various forms of H5 HA DNA vaccines, the HA antigens expressed from 293T cells [45], [46] were treated with PNGaseF (New England BioLab, Beverly, MA) [47], [48], [49]. Briefly, the HA proteins were first denatured at 100°C for 10 min in glycoprotein denaturing buffer and then chilled on ice. Following addition of G7 reaction buffer, the deglycosylation enzyme cocktail was added and incubated reaction at 37°C for 4 hours. Either mock-treated or deglycosylated HA samples were subjected to SDS-PAGE and Western blot analysis was performed as described above.

### ELISA (Enzyme-linked immunosorbent assay)

ELISA was conducted to measure HA-specific antibody (IgG) responses in immunized rabbits and mice. The 96-well flat-bottom plates were coated with 100 µl of ConA (50 µg/ml) for 1 hour at room temperature, and washed 5 times with PBS containing 0.1% Triton X-100. Subsequently, the plates were incubated overnight at 4°C with 100 µl of transiently expressed HA antigen at 1 µg/ml. After being washed 5 times as above, the plates were then blocked with 200 µl/well of blocking buffer (5% non-fat dry milk, 4% Whey, 0.5% Tween-20 in PBS at pH7.2) for 1 hour. After five washes, 100 µl of serially diluted rabbit or mouse serum was added in duplicate wells and incubated for 1 hour. After another set of washes, the plates were incubated for 1 hour at 37°C with 100 µl of biotinylated anti-rabbit or anti-mouse IgG (Vector Laboratories, Burlingame, CA) diluted at 1∶1000 in Whey dilution buffer (4% Whey, 0.5% Tween-20 in PBS). Then, 100 µl of horseradish peroxidase-conjugated streptavidin (Vector Laboratories) diluted at 1∶2000 in Whey buffer was added to each well and incubated for 1 hour. After the final washing, the plates were developed with 3,3′,5,5′ Tetramethybenzidine (TMB) solution at 100 µl per well (Sigma, St. Louis, MO) for 3.5 minutes. The reactions were stopped by adding 25 µl of 2 M H_2_SO_4_, and the plates were read at OD 450 nm. The end titration titer was determined as the highest serum dilution that has an OD reading above twice of that from the negative control serum.

### Preparation of the influenza A viruses stocks

Influenza A viruses of the H5N1 A/HongKong/483/97 (H5N1), A/Viet Nam/1203/04 (H5N1), A/Anhui/1/2005 (H5N1), and A/Indonesia/5/2005 (H5N1) were grown in the allantoic cavity of 10-day-old embryonated hen eggs at 37°C for 26 to 40 h. Allantoic fluid pooled from multiple eggs was clarified by centrifugation and frozen in aliquots at −70°C. The 50% egg infectious dose (EID50) for each virus stock was calculated by the method of Reed and Muench following serial titration in eggs. All experiments with HPAI viruses were conducted under Biosafety Level 3 containment.

### Hemagglutination-inhibition (HI) assay

HI assays were performed by standard methods [50]. Briefly, the assay was performed using, 0.5% v/v fowl or horse [30] red blood cells, 4 HA unit of reference H5N1 virus (A/HongKong/483/97, A/VietNam/1203/04, A/Anhui/1/2005, A/Indonesia/5/2005) and specific sera treated with receptor destroying enzyme. The HI titer was defined as the highest dilution of the serum able to inhibit hemagglutination.

### Microneutralization (MN) assay

MN assays was performed as described previously [51]. In brief, influenza virus containing 100 TCID_50_ was incubated with equal volume of two-fold dilutions of the specific heat-inactivated serum overnight at 37°C in a 5% CO2 humidified atmosphere for 1 hr. After the incubation, 100 µl virus-serum samples were added to a 96-well plate containing Madin Darby Canine Kidney (MDCK) cell monolayer and incubated for 5 days at 37°C and 5% CO2. The microneutralization titer was defined as the highest dilution of serum that neutralized 100 TCID_50_ of virus in MDCK cell [52], [53] cultures (as detected by the absence of cytopathic effects). The MN assays were conducted in two different labs: 1) National Microbiology Laboratory, Public Health Agency of Canada, and 2) Beijing Institute of Microbiology and Epidemiology, according to the viruses available.

### Pseudotyped virus (PV) assay

The recombinant lentiviral vectors expressing a luciferase reporter gene were produced as previously described [28], [54], [55]. To produce H5N1 pseudotyped viruses, 293T cells [45], [46] (5×10^6^ cells plated the day before) were transfected with 13.43 µg of pNL 4-3.Luc.R-E- (NIH AIDS reference and reagent program), 1.2 µg of H5-HA-wt DNA vaccine plasmid and 0.3 µg of N1-NA plasmid using 75 µg of polyetheleneimine transfection reagent. Supernatants were harvested 48 hours later, frozen at −80°C and then standardized by infectivity in 293A cells using a luciferase-based TCID50 measurement. For neutralization assays, serum samples (5 µl) were heat inactivated at 56°C for 30 minutes, then three-fold serially diluted in culture medium in flat-bottomed microtiter plates. Pseudotyped H5N1 virions were then added to the plates at 200 TCID50/well and incubated for 1 hour at 37°C. After incubation, 293A cells were trypsinized and added to each plate at a dilution of 1×10^4^ cells per well. Following a 48-hour incubation, plates were developed using a luciferase assay system (Promega). Values averaged from triplicate wells were then used to determine IC50 based on wells that displayed 50% reduction in infection as compared to control wells containing virus plus pre-immune sera.

## Supporting Information

Figure S1 **Western blot analysis of potential N-linked glycosylations of HA protein expressed by differently designed H5-VN (A/VietNam/1204/04) DNA vaccines: H5-VN.wt, H5-VN.tPA, H5-VN.dTM or empty vector, transfected 293T cell lysate or supernatant, with (F) or without (U) PNGase F treatment.**
(TIF)
