# Supplementary figures and images for: Polyvalent DNA Vaccines Expressing HA Antigens of H5N1 Influenza Viruses with an Optimized Leader Sequence Elicit Cross-Protective Antibody Responses
Source: PLoS One. 2011 Dec 21;6(12):e28757. doi: 10.1371/journal.pone.0028757 (PMC3244406; doi:10.1371/journal.pone.0028757)

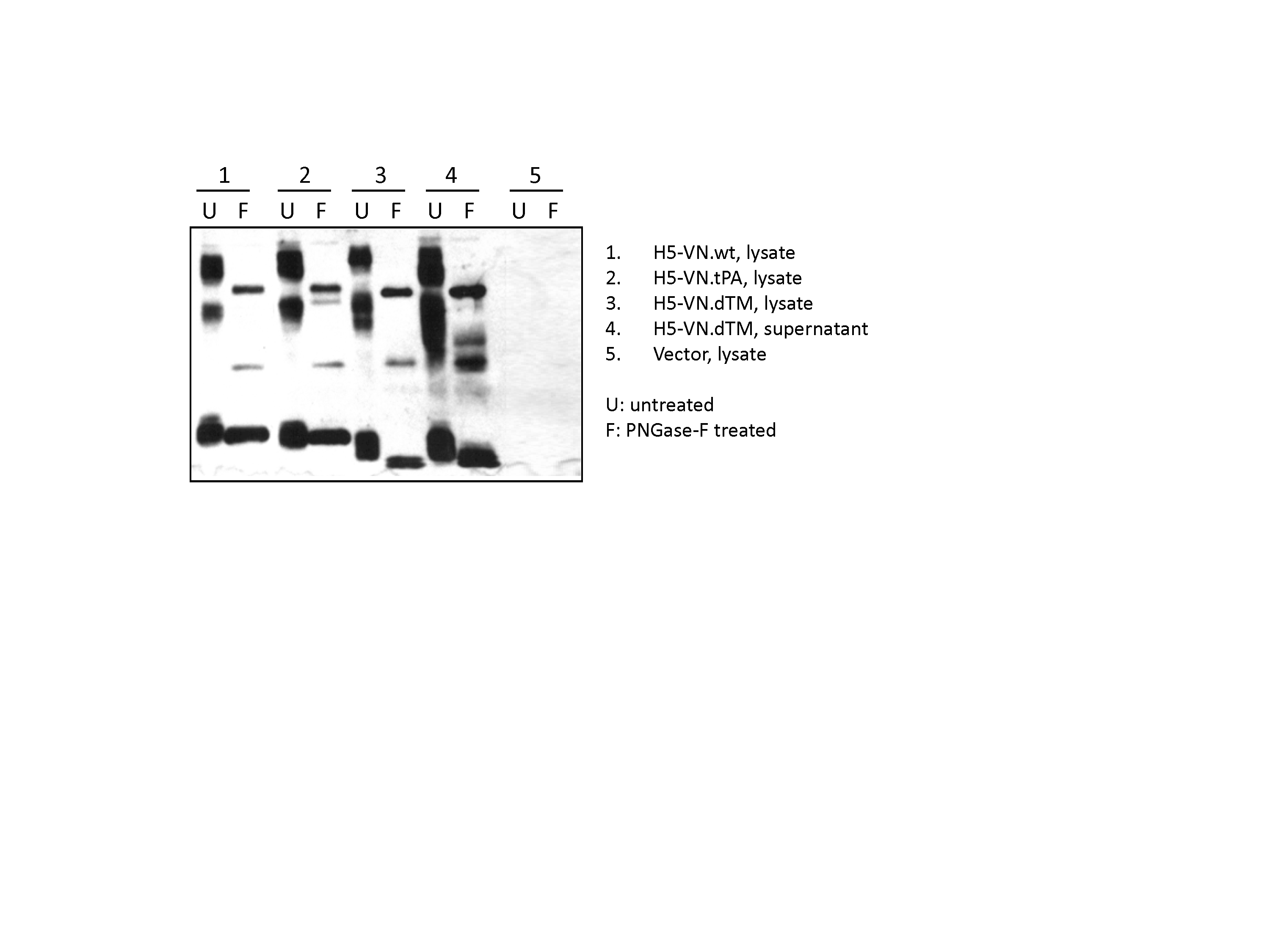

Supplement: Figure S1 — Western blot analysis of potential N-linked glycosylations of HA protein expressed by differently designed H5-VN (A/VietNam/1204/04) DNA vaccines: H5-VN.wt, H5-VN.tPA, H5-VN.dTM or empty vector, transfected 293T cell lysate or supernatant, with (F) or without (U) PNGase F treatment. (TIF) [file pone.0028757.s001.tif]
